# Making open data work for plant scientists

**DOI:** 10.1093/jxb/ert273

**Published:** 2013-09-16

**Authors:** Sabina Leonelli, Nicholas Smirnoff, Jonathan Moore, Charis Cook, Ruth Bastow

**Affiliations:** ^1^Egenis & Department of Sociology, Philosophy and Anthropology, Byrne House, St Germans Road, Exeter EX4 4PJ, UK; ^2^Geoffrey Pope Building, Biosciences, University of Exeter, Stocker Road, Exeter EX4 4QD, UK; ^3^Warwick Systems Biology Centre, Senate House, University of Warwick, Coventry CV4 7AL, UK; ^4^School of Life Sciences, Gibbet Hill Campus, University of Warwick, Coventry CV4 7AL, UK

**Keywords:** Data sharing, databases, metabolomics, open data, proteomics, publication, repositories, transcriptomics.

## Abstract

Despite the clear demand for open data sharing, its implementation within plant science is still limited. This is, at least in part, because open data-sharing raises several unanswered questions and challenges to current research practices. In this commentary, some of the challenges encountered by plant researchers at the bench when generating, interpreting, and attempting to disseminate their data have been highlighted. The difficulties involved in sharing sequencing, transcriptomics, proteomics, and metabolomics data are reviewed. The benefits and drawbacks of three data-sharing venues currently available to plant scientists are identified and assessed: (i) journal publication; (ii) university repositories; and (iii) community and project-specific databases. It is concluded that community and project-specific databases are the most useful to researchers interested in effective data sharing, since these databases are explicitly created to meet the researchers’ needs, support extensive curation, and embody a heightened awareness of what it takes to make data reuseable by others. Such bottom-up and community-driven approaches need to be valued by the research community, supported by publishers, and provided with long-term sustainable support by funding bodies and government. At the same time, these databases need to be linked to generic databases where possible, in order to be discoverable to the majority of researchers and thus promote effective and efficient data sharing. As we look forward to a future that embraces open access to data and publications, it is essential that data policies, data curation, data integration, data infrastructure, and data funding are linked together so as to foster data access and research productivity.

## Introduction: why is open data important in plant science?

Scientists have long developed effective practices for the dissemination of selected datasets to accompany specific claims which typically involve the publication of papers in widely available journals. Publishing data in this way, however, means that only a small fraction of the data produced in any one laboratory is made publicly accessible and that the selection of data for publication depends solely on their value as evidence for the claims made by the authors of the paper in question. This is particularly problematic in the wake of increasingly large-scale datasets and research communities in biology; the multi-disciplinary and geographically dispersed nature of international research networks; and the strong scientific and political support for the idea that data generated in one laboratory can and should be reused for a variety of different purposes by multiple researchers. Within plant science, technologies such as next generation sequencing (NGS) are lowering the barriers to research in economically important plants (Brenchley *et al.*, 2012; IBGS 2012) and allowing in-depth studies of model species (as in the case of the *Arabidopsis* 1001 genome project; http://www.1001genomes.org/). These advances provide substantial opportunities for advancing scientific understanding and funders and researchers agree that research groups around the globe should widely and freely disseminate data for future use. Plant scientists are thus increasingly encouraged, and often required, to donate data to open access databases, regardless of whether or not these data are associated with a publication (for instance, in the UK by the BBSRC data management policy; http://www.bbsrc.ac.uk/web/FILES/Policies/data-sharing-policy.pdf; and in the US by the NSF data sharing policy http://www.nsf.gov/bfa/dias/policy/dmp.jsp); and to make use of these databases in order to boost their research and speed up discovery (Ball and Duke, 2012). A recent report by the Royal Society specifically points to the urgent need for ‘intelligent’ data access (Royal Society 2012), which involves investing resources, time and effort in making data publicly available, findable, interpretable, reusable, and citable. The drivers for this requirement include several key objectives for the advancement of scientific research in the 21st century: increasing the transparency and reproducibility of research; speeding up research by facilitating cross-consultation and comparison among existing datasets; making the best of available resources by reducing duplications in the research process; introducing new methods for discovery, based on the partly automated mining of large datasets; and improving teaching and collaborative research in both the developed and the developing world, by making data produced through expensive and/or unique instruments and materials widely available for query and analysis.

Despite the clear demand for data-sharing and the strength of the motivations for it, its implementation is still limited (Alsheikh-Ali *et al.*, 2011). This is because data-sharing raises several unanswered questions and challenges to current research practices. Firstly, plant scientists have to deal with a variety of data types including ‘omics’ data, imaging data (varying in scale from field phenotyping to cell biology), modelling results, natural variation and diversity data. The wide variety of datasets and types makes it very hard to make generic decisions on whether it is feasible and useful to store and disseminate all of these data. It is also unclear how decisions should be made about which datasets are most useful for dissemination or which types of data should have priority when setting up databases and curatorial standards, particularly given that standards set today are likely to change in the future. For example, The *Arabidopsis* Information Resource (TAIR; http://www.arabidopsis.org), which was established in 2000 as a portal centred on a single genome (Columbia 0 accession) and associated data, was not set up to deal with the current data deluge that includes thousands of different genomes and epigenetic data. The community is thus planning a new *Arabidopsis* Information Portal (IAIC 2010, 2012) which will build upon the expertise of TAIR, provide additional layers of functionality and tap into broader initiatives to support plant data sharing in the US (http://www.iplantcollaborative.org/) and in the EU (http://www.elixir-europe.org/); but in the meantime, much of the data produced on *Arabidopsis* does not have an obvious home. Secondly, when it comes to the infrastructure, support, and accountability of these data there is no single answer to who should maintain structures to host data and support them financially in the long term (Bastow and Leonelli, 2010); how responsibilities and related duties to data curation, such as the authorship of data and the efforts spent in posting them online, need to be allocated and rewarded within the scientific system; how such responsibilities need to be policed or enforced, and by whom (universities, institutions, publishers, funding bodies, and national governments); and how to go from efficient dissemination to intelligent reuse. The ways in which those issues will be discussed and tackled in the future is crucial to the development and survival of the plethora of databases and resources that are currently being established to handle the storage, dissemination and analysis of plant data, such as Ensemble Plants (http://plants.ensembl.org/index.html), CoGE (http://genomevolution.org/CoGe/), and the bio-array resource for Plant Biology (http://bar.utoronto.ca/welcome.htm).

In this paper, we contribute to these discussions by reviewing some of the challenges encountered by plant researchers at the bench when generating, interpreting, and attempting to disseminate their data. The opportunities and difficulties involved in sharing data of different types (particularly transcriptomic, proteomic, and metabolomic data) are considered and the strategies that can be used by plant scientists to share these data efficiently and effectively in the future are assessed, thus complying with funding requirements while at the same time fostering research productivity.

## Storing and sharing high throughput or large-scale data

Access to high throughput methods now allows experimenters to collect much more data than is needed for a specific experiment. Only a very small proportion of all the data produced as result of experimentation are directly used in publications or analysed to answer the question at hand. Therefore there is a vast amount of data available that needs to be stored and made available to be mined for other purposes. However, it is unclear how researchers can disseminate and utilize the wealth of data that are produced via the diverse techniques in use across plant biology. Examples of such variety include transcriptomics data from arrays or RNA sequencing, genome sequencing via NGS, and proteomics and metabolomics data whose characteristics are reviewed in this paper. In addition, plant scientists also have to grapple with high throughput plant phenotyping, which is making it possible to collect yet another wide range of data relating to plant growth, development, and environment; and with the many other types of evidence generated by other fields, including cell biology images, mathematical models, photographs, movies, and software. Those involved in the numerous projects that are digitizing old material, such as journal articles, figures or herbarium samples, are also facing a similar data dilemma. In confronting this complex and rich landscape of potential data sharing, it is important to note that not all data are equal: some are easier to share, reuse, deposit, and integrate than others. This typically depends on the variability of data, the materials (specimens, tissue cultures, etc) and experimental methodologies used, and the availability of standards for formatting, annotating, and depositing data.

*Sequence data* are relatively easily to analyse, annotate, and store as ultimately they consist of the four discrete units of DNA code ‘A,G,C,T’, whose format is fairly standard and whose notation is easily digitized. Repositories and databases housing genome sequence data are well established and all almost all of them are publically available, for example, GenBank and TAIR. The same is generally true for *transcriptomics data* obtained through experiments that measure genome-wide gene expression in tissues, organs or organisms as a means to observe responses to environmental or endogenous perturbations. Such experiments employ measurement technologies including microarrays and high-throughput transcript sequencing that allows researchers to generate relatively large raw datasets, showing the expression of all annotated genes in multiple samples according to the experimental conditions. These comprehensive raw data are subsequently reduced through analysis and by synthesis with previously published data, to generate focused biological insights which are then reported in peer-reviewed publications. Microarrays, and to an increasing extent transcript sequencing, are now mature technologies, with accepted data formats and statistical methods for analysis (although there is active methods development) and community standards for reporting metadata (Rogers and Cambrosio, 2007). To ensure repeatability and openness, raw data are routinely made accessible, often as a condition of publication of the reduced results in a journal. Internationally agreed community standards for reporting such experiments (e.g. Minimum Information About a Microarray Experiment—MIAME; Brazma *et al.*, 2001) have been developed to ensure broad comparability between datasets, and to allow datasets from disparate laboratories to be readily collated and queried in public databases. Such raw and processed data from expression experiments, and the associated metadata meeting MIAME standards, are lodged in the public repositories Gene Expression Omnibus (GEO; Barrett *et al.*, 2011) and Array Express (Rustici *et al.*, 2013). Submission of the data typically requires the raw files to be bundled with a spreadsheet, XML or other structured document that contains the processed data and metadata. Publication of an accession number from either of these databases is generally a requirement for publication of gene expression results in a peer-reviewed journal. Once in the public repositories, these data are then available to all researchers and, in some cases, subsets of the public corpus are aggregated in further databases, for instance, to show expression of genes in a particular organism under a wide range of experimental conditions.

Compared with transcriptomics data, *proteomics data* are more variable and thus more difficult to annotate and share. Proteomics data are largely derived from mass spectrometry either by analysis of spots picked from gels or from liquid chromatography (LC) followed by mass spectrometry (MS) or MS experiments. Proteomics datasets include information on peptides/proteins identified in various tissues, organelles or through a quantitative comparison of protein/peptide abundance in different samples on a more global scale. Comparative data might be the result of methods that use tags to label peptides from different samples (or they might be derived from label-free comparisons. Importantly, interpretation of MS-based proteomics is dependent on the data-mining algorithms used and the identification of peptides by database searching involves some uncertainty. Therefore, retrospective analysis using new algorithms requires access to both raw and processed data which, in turn, means that raw data should be made available in a format suitable for analysis by open source software. There are a number of such formats in use currently including mzXML and mzDATA. The analysed data consist of lists of identified peptides and their sequences, a score providing information on the confidence of identification, peptide/protein abundance, and the mass/charge and MS/MS spectra of the peptides. Plant proteomic data can be found in databases such as PRIDE (http://www.ebi.ac.uk/pride), the Plant Proteome Database (http://ppdb.tc.cornell.edu/), AT Chloro (http://www.grenoble.prabi.fr/at_chloro/), and the plastid protein database (http://www.plprot.ethz.ch/). SUBA (http://suba.plantenergy.uwa.edu.au/) provides a subcellular localization database for *Arabidopsis* proteins and various other *Arabidopsis* proteomics resources are listed on the GARNet website (www.garnetcommunity.org.uk) and TAIR (http://www.arabidopsis.org/portals/proteome/index.jsp). Individual laboratories curate most of these databases. In general, they tend to contain information (including mass spectra) of identified peptides but not comparative proteomics data (i.e. comparison of protein abundance in different samples). PRIDE provides an interface that enables users to inspect and mine uploaded data, as well as a detailed description of how researchers should format and submit data as per the standards developed by the Proteomics Standards Initiative (PSI). Although community-agreed standards for reporting and publishing proteomics data do exist, MIAPE (the Minimum Information about a Proteomics Experiment; http://www.psidev.info/node/91), they are not as well established or utilized as those for transcriptomics.

*Metabolomic data* are even more complex to produce and interpret compared with proteomic data and thus harder to curate and house. The term metabolomics is used here to describe experiments that attempt to capture as much of the chemical composition of a tissue extract as possible and to compare it between samples. This is difficult to achieve because, unlike nucleic acids and proteins, the diversity of small molecules means that no one extraction and analytical technique can detect everything. Added to this, and again unlike transcriptomics and proteomics research, no plant has a reference metabolome against which comparisons can be made and no exact figure can be put on the number of small molecules in *Arabidopsis*. Metabolomics data are diverse in the collection techniques used and their degree of complexity. At one end of the scale, metabolite fingerprinting by spectroscopic techniques such as infrared (IR), Raman, and nuclear magnetic resonance (NMR) spectroscopy produce easily stored and read files which an analytical biochemist with suitable skills can easily interpret. At the other end is mass spectrometry (MS), the most widely used approach and one that usually requires prior chromatographic separation of analytes by gas chromatography (GC) or liquid chromatography (LC). Interpretation of MS-based metabolite profiling presents a formidable challenge. Many features detected in these experiments cannot be identified or verified without standard compounds (of which there is a limited selection) or further purification and detailed analysis. In a typical LC-MS based metabolomics experiment, only 10–20% of features can be firmly identified. For this reason, metabolomics has arguably not lived up to its original promise in delivering an enhanced understanding of plant function. Indeed, most experiments report an array of compounds that could have been measured quantitatively by well-established techniques in analytical biochemistry.

The difficulties in adequately curating and interpreting metabolomics data might also account for the scarcity of plant metabolomics data currently available in public databases. Some of the existing resources feature data derived from specific experiments which database users can retrieve and upload to their own computers. For instance, Metabolome Express (www.metabolome-express.org) is focused on GC-MS metabolite profiling and includes tools for the extraction of information from raw data files as well the ability to carry out comparative analyses. Other metabolomics databases, like the *Arabidopsis* Metabolomics Consortium (http://plantmetabolomics.vrac.iastate.edu/ver2/index.php) and the Platform for Riken Metabolomics (PRIMe, http://prime.psc.riken.jp/) are even more project-specific. Further, there are a number of databases storing NMR spectra and MS spectra that can be used for aiding compound identification, for example: Golm Metabolite Database (http://gmd.mpimp-golm.mpg.de/); Platform for RIKEN Metabolomics (http://prime.psc.riken.jp/); MassBank (http://www.massbank.jp/?lang=en); Biological Magnetic Resonance Data Bank (http://www.bmrb.wisc.edu/); and Human Metabolome Database (http://www.hmdb.ca/)—whilst the Plant Metabolomic Network (http://www.plantcyc.org/) provides information on plant metabolic pathways. To remedy the difficulties in disseminating metabolomics data, various standards for reporting this type of data have been proposed over the years which provide useful pointers on the information required. A recent example consists of spreadsheet templates for reporting metabolite profiling data (Fernie *et al.*, 2011). However, journals rarely give specific guidance on how to format metabolomics data or in which public databases researchers should deposit raw data. Metabolite profiling data are often presented in publications as supplementary data files on journal web sites. These often do not provide sufficient information for the reader to assess the reliability of identification and are sometimes limited to lists of compounds identified without further information. Given their complexity and the potentially large size of raw data files, supplemental information is thus not the most suitable place to store metabolomics (or even proteomics) data. It also has the added disadvantages of uncontrolled presentation and lack of discoverability via computational searches.

Like the -omic datasets considered in this section (summarized in Table 1), much of the data produced in plant science are typically the results of focused experiments where data are produced to answer a specific question. As we illustrated, making these data reusable by scientists who were not directly involved in their production is no easy task. Transcriptomics constitutes one of the most straightforward cases of data dissemination. These data are typically derived from large-scale experiments and their format is highly standardized with little variation; their sharing and reuse is facilitated by the existence of community-agreed standards for annotation and deposition and there are numerous globally available databases and resources tailored for the deposition and analysis of these data. By contrast, metabolomics data are highly variable, generally produced by a single group or researcher, and difficult to standardize and curate. To retrieve, reanalyse, and reuse metabolomics data effectively at a level comparable with transcriptomics data will therefore require investment in adequate skills, resources, and time for their curation, as well as the establishment of formatting and annotation standards. The level of effort and investment required to achieve this is likely to be much greater than that required for sequence-based data, but it will be essential in order to ensure that metabolomics data can be reused in other investigations and comparative studies.

**Table 1.** Comparison of features of transcriptomics, proteomics and metabolomics data of particular relevance for reuse

	Transcriptomics	Proteomics	Metabolomics
Instruments used for data production	Next generation sequencing microarray experiments, transcript sequencing	Mass spectroscopy (spots picked from gels or from LC-MS/MS experiments)	Metabolite fingerprinting by spectroscopic techniques such as IR, Raman and NMR; or mass spectroscopy preceded by liquid or gas chromatography
Typical data producers	Large research networks or apposite institutes (e.g. TGAC)	Research groups, medium-sized projects	Small groups or individuals
Specific challenges to interpretation and reuse	Importance of reporting environmental conditions of data generation (now largely standardized)	Variety of sources for information on peptides/proteins. Also, interpretation depends on data mining algorithms used	Varieties of small molecules involves multiple measurement techniques, i.e. complex and large supplementary data

## Strategies for data release and accessibility: where to go?

It is essential that plant data are made available in ways that make them as reusable as possible on as large a scale as possible. In this section, this criterion is used to evaluate the usefulness and applicability of three types of mechanisms currently used for data dissemination by the plant science community: journals, university repositories, and community/project databases.

### Scientific journals

The majority of publishers have specific guidelines for the annotation, formatting, and depositing of sequence data that are published in the main body of a paper. They all require datasets to be available for review in their entirety after submission and to be deposited in a community-standard publically available database after publication, such as ArrayExpress or GEO. Further, they all expect microarray data to comply with MIAME guidelines. The vast majority of international journals in plant science also impose some basic data-sharing principles and require authors to use internationally agreed nomenclature for data classification (which varies among species) where applicable. However, there are substantial variations in how strictly journals abide by these guidelines; and in the specific requirements and support offered for data-sharing. For instance, *The Journal of Experimental Botany* explicitly encourages authors to submit gene function data to TAIR, and *Plant Physiology* has made it a condition for the submission and acceptance of papers. *The Plant Cell*, by contrast, does not specify through which database data should be made available, but it does require that sequence data be in an easily readable format and allows highlighting to display certain features. *Science* requires all datasets to be MI-compliant if applicable; whilst *Nature* specifies submission to a community-endorsed public repository for DNA, RNA, and protein sequence and microarray data. Only one of the major plant science journals provides explicit instructions on how to format metabolomics datasets for inclusion as supplementary materials; some, but not all, journals advise authors to follow the MIAPE standard for proteomic datasets (http://www.psidev.info/MIAPE). This diversity of guidelines makes it difficult for authors to understand how best to format and curate data for the purposes of public consultation and prospective reuse.

**Fig. 1.**
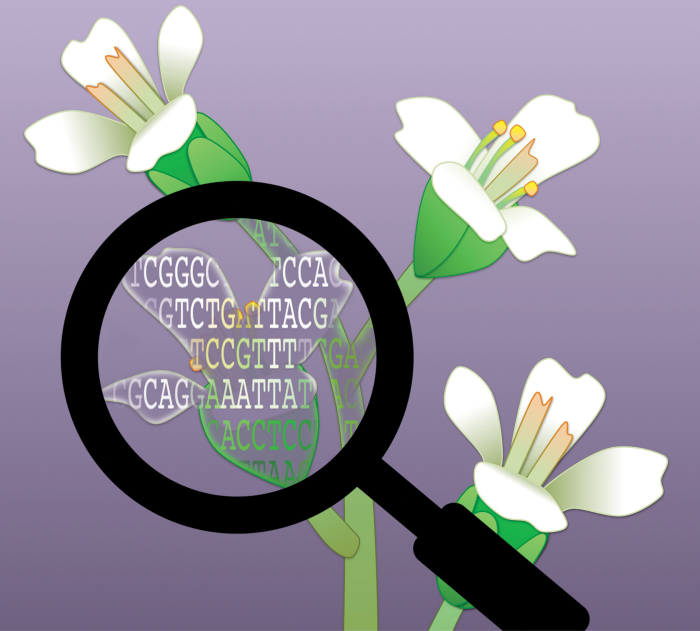
Data dissemination is key to the development of plant science.

Another problematic area for data access via the traditional publishing model is the use of supplementary information (SI), which was created to deal with data that could not fit within a paper in journals that are limited to print space, such as *Nature* and *Science*, and to allow publication of large datasets or data in print-inaccessible media such as videos. This seems to have been successful, as SI submissions have increased exponentially in recent years. However, SI is hard to access and reuse, as it is often only available in.pdf format and not.xml or.html format, which makes it hard to index and search. In addition, SI may not undergo the same rigorous review process as data in the main body of the paper, resulting in a lack of quality control of the associated metadata. This is partly dependent on each journal’s policies, and partly on the difficulty involved in finding referees to assess data quality (which constitutes yet another demand on referee’s time). There are also concerns that the unlimited space provided by SI can be utilized as a mechanism by referees continually to ask for additional experiments and datasets. Despite its original usefulness, there are well-founded fears from researchers and publishers alike that SI could rapidly turn into a ‘data dump’ which has no clear guidelines for curation, review/refereeing, and no mechanism to extract and reuse data easily. An obvious solution to this problem is to remove SI, if data are really essential to understanding and evaluating claims in a paper, they should be included in the paper itself rather than being singled out as SI. This option is currently being undertaken by the *eLIFE* journal (http://www.elifesciences.org/) and the *Journal of Neuroscience* and it will be interesting to follow the outcomes of this experiment.

The extent to which journals can support data-sharing is related to the support offered by publishing houses towards the funding and development of databases through which the datasets used in research articles can be made discoverable. It is standard for many journals either to link to the data entity via a DOI or to re-direct readers to an existing, publicly sponsored repository such as the Protein Databank (http://www.wwpdb.org/), GenBank (http://www.ncbi.nlm.nih.gov/genbank/), PubChem (http://pubchem.ncbi.nlm.nih.gov/), and GEO. In addition, some journals have integrated datasets within the paper via APIs or webservices, for example, data from the PANGAEA database (http://www.pangaea.de/) or genome data provided by TAIR. Connecting data in this way helps to increase data discoverability, keeps the data linked and in context with the research paper it is associated with, and improves online readability. However, this approach for integrating and linking to data is only feasible for data that are stored in well-established repositories. It is not possible for datasets without an established home or small sets of data, such as those regularly used to produce a figure or table within in a paper, to be accessed in such a way. Such data are therefore not available to users and cannot be reused.

A clear strategy for the role of journals and publishers in data access and release is lacking at present, but answers will need to be found if publishers and researchers are to adhere to open access policies such as the one currently endorsed by Research Councils UK (RCUK; http://www.rcuk.ac.uk/documents/documents/RCUKOpenAccessPolicy.pdf). One of the aims of the RCUK policy is ‘for all users to be able to read published research papers in an electronic format and to search for and re-use (including download) the content of published research papers, both manually and using automated tools (such as those for text and data mining), provided that any such re-use is subject to full and proper attribution.’ It might be viewed as unfeasible for journals to be directly involved in data storage and publication, given the related costs and their lack of expertise in data curation. Yet it is not clear why publishers should not be responsible for the maintenance of a database(s), in which essential data used as evidence in published papers is stored and accessible so as to reach the aim stated above.

One increasingly popular solution to the data store problem is the use of generic data stores such as Dryad (http://www.datadryad.org/), Figshare (http://figshare.com/), and DataOne (http://www.dataone.org/), which is strongly supported by publishers in the Company of Biologists as well as the Nature Publishing Group. Another possible solution resides in the rise of data-only journals such as *Ecological Archives*, *ZooKeys*, *F100Research*, *GigaScience*, *Database* and, most recently, *Scientific Data*. These journals facilitate data reuse by providing access to easily retrievable, high-quality, curated datasets. They also publish papers documenting innovations in standards, methods, and strategies for data dissemination, thus fostering the development of appropriate resources and tools for data reuse. They differ from other journals insofar as the publication of datasets is not tied to a specific interpretation of their scientific significance (accepting the Open Data motto that ‘the best use of your data will be thought up by someone else’). They provide the opportunity for data producers and curators to be credited for their work; and thus enhance the perception of research data as a research output in itself. Despite these advantages, it needs to be noted that the difference between a data journal and a well-curated data repository is not clear but, in both cases, it is essential to have adequate metadata detailing the provenance of data. Data journals seem to be particularly useful as vehicles for the many types of data that are not yet hosted in large repositories. This is arguably why data journals are being founded on a weekly basis. However, it is not obvious to what extent ‘journal publication’ format can ensure interoperability and comparability of datasets. In order to facilitate integration and comparison among data types, the best solution still seems to be to contribute directly to a publically available database that is based on agreed community standards, where available.

### University repositories

In 2011, RCUK issued a set of Common Principles on research data policy, under which data are to be made openly available with as few restrictions as possible in a timely and responsible manner. The interpretation of these principles varies across UK funding bodies. An example is the expected duration of data storage: the Art and Humanities Research Council (AHRC) requires access for three years, the Biotechnology and Biological Sciences Research Council (BBSRC) and the Medical Research Council (MRC) state a period of 10 years, whilst the Engineering and Physical Sciences Research Council (EPSRC) expects data to be preserved for a minimum of 10 years. In terms of data management and access, most UK funders request a data-management and sharing plan in their grant whilst others such as EPSRC expect the UK institutions that they fund to develop a data policy and roadmap. These expectations, along with the fact that some funders such as BBSRC state that ownership of data resides with the investigators and their institutions, has resulted in an emphasis on universities as essential contributors to data storage. Some of the top universities in the UK, such as University College London, are thus developing institutional data repositories, to which staff are increasingly expected to contribute. The growth of institutional repositories is not limited to the UK: in the US, the National Science Foundation (NSF) and the National Institute of Health also require a data management plan and numerous data storage projects are popping up across the US, such as the large-scale Data Conservancy housed at the John Hopkins University.

The use of university repositories is understandable, given that universities constitute the first port of call for providing substantial support to researchers wishing to share data and that a localized approach can arguably best tackle specific research requirements. However, for this to be an effective solution it requires appropriate infrastructure and support, such as adequate *training in data management*, perhaps via university libraries and specialized departments responsible for providing research support (see the special issue of *Nature* on reinventing libraries; Vol. 495, 28 March 2013), and adequate *IT provision*, including servers and technical assistance with data curation, formatting, and the development of project-specific databases. It also requires that staff, and thus research projects, are allocated the appropriate time to allow for data to be curated to a level that would allow sharing and reuse, perhaps by inserting data-sharing as a component of individual workload assessments. This, in turn, will necessitate a shift in the system of *credit attribution*, so that dat-sharing is seen as a valid research output and can be used in promotion applications. Finally, universities need to provide *clear guidelines* concerning what is expected of staff when it comes to data publication and data access. This will help to ensure that depositing data in institutional repositories has no negative consequences for the reuse of data by others and that the data are publically available and easily discoverable. It is also essential that university repositories be structured in such a way that they are *interoperable* with existing and highly visible international databases specialized in the relevant scientific fields and data types. In addition, such repositories must have sufficient financial support to provide *long-term* staffing to maintain the repository, train users, and allow extensibility for future needs.

One possible downside of relying on universities as the main administrators of data storage and dissemination is that a single institution has to provide capacity and capability to store the wide range of data types generated by its entire staff. This would be a huge and onerous task and it seems unlikely that one university would contain all the relevant expertise and funding to curate and house all the data it generates, as there are too many field-dependent and data type-dependent variables. A more sensible solution is for universities to stipulate that, where possible, data are stored in internationally recognized databases that specialize in that data type. This removes an extra layer of complexity in searching for and locating data, prevents duplication of efforts, and promotes best use of investments. In cases where internationally recognized databases do not exist, then the university repository can provisionally function as the data store. However, this would still be a sizeable task and, in many cases, is likely to require additional sources of income to provide adequate and long-term support.

If guiding principles and strategies similar to those outlined above were imposed on institutional repositories, then they could become a key layer in supporting data accessibility and reuse. However, if they are not regulated at some level, institutional repositories risk becoming inward-looking databanks that only serve the interests of the institution that established them, rather than assisting the wider scientific community.

### Community/project-specific databases

These are databases that have been developed by a specific group of researchers for the purpose of disseminating their own results (hence ‘project-specific databases’) or by a network of researchers in order to provide better access to a given dataset or data-type (hence ‘community databases’). This type of approach is supported by funding bodies such as BBSRC and NSF, which take the view that data-sharing should be led by the scientific community and driven by scientific need. Other funders, such as the Natural Environment Research Council in the UK, take a different view and provide funded data centres that are not focused on a specific project or linked to a scientific community.

Some of the most successful forms of data release have come in the form of databases developed and curated by scientists themselves with the support of public funding. For example, NASC arrays, TAIR, and Gramene in the plant sphere and VectorBase, Wormbase, and FlyBase outside the plant world. These databases operate on different levels of data granularity. Some focus on a single data type, whilst others act as aggregators to allow cross-query and data integration. Examples of the latter include the Bio-Analytic Resource for Plant Biology, based at the University of Toronto, that encompasses a variety of tools to work with functional and other data on plants—such as the *Arabidopsis* electronic Fluorescent Pictograph (eFP) Browser (Winter *et al.*, 2007). This integrates large-scale datasets such as transcriptomics into a pictorial representation based on plant development to aid interpretation. Genevestigator integrates transcriptome data from various sources (including NASC) into a visual /graphical representation and is another good example of a resource grown from existing project-based public data (Zimmerman *et al.*, 2004). Genevestigator is widely used and cited, and its basic version, which includes gene expression data from several species including barley, rice, wheat, *Arabidopsis*, and soybean, is accessible online for free. However, the full version with additional data analysis and visualization tools requires a paid subscription.

When the development of such resources is examined closely, a common pattern emerges: all of them start when one single research project finds a need for a particular resource, commits to developing that resource, and subsequently shares it with a wider community. This ‘bottom-up’ development is a well-documented way to design public infrastructure, within which the emergence of resources is organic rather than planned. It is developed to meet immediate scientific needs and can then be applied in broader contexts. Examples of this broadening of scope can be seen in the recent extension of the Ensembl platform to incorporate plants and other organisms, from its original scope and focus on human biology, and on the extension of the FlyMine query platform to the InterMine all-organism platform.

An example of a database born out of a specific research effort is that generated by the PRESTA (Plant Responses to Environmental Stress in *Arabidopsis*) project. This project, which is funded by BBSRC and EPSRC and involves the Universities of Warwick, Essex, and Exeter, generated a number of large datasets from high-throughput transcriptomics time-series experiments of plants’ responses to a variety of environmental stresses (Breeze *et al.*, 2011; Baxter *et al.*, 2012; Windram *et al.*, 2012; Hickman *et al.*, 2013), undertook a systematic literature review of gene regulatory interactions in *Arabidopsis*, and carried out a number of focused experiments to test interactions between plant transcription factors and promoters, with the aim of elucidating regulatory networks. The project also developed a range of models and subsequently estimated datasets from the application of the modelling approaches. It was clear from the onset of the project that no obvious public platforms were available to integrate literature-based gene interactions, time-series expression data, and modelling results, in an intuitive way to allow novel biological discovery. A database was therefore built to allow the disparate experimental datasets and models to be queried in the context of the information gathered from the published literature. This Interactions, Domains, Experiments, and Annotations (IDEAs) database was designed to be organism- and project-agnostic from the start, and so is being made public as a resource to help meet the needs of other similar projects in future.

Databases such as IDEAs are key to the ‘intelligent reuse of data’ outlined by the Royal Society (Royal Society, 2012). However, due to the ‘niche status’ they generally do not attract funding and are not linked to or supported by more generic repositories. Therefore, unless the data stored within these project databases is associated with a commonly studied organism or a ubiquitous measurement technology, most researchers will not be aware of it. Individual researchers, communities, and institutions, therefore, all need to work together, either to support the discoverability and long-term persistence of unique and genuinely useful databases or to ensure that such databases move data outwards into more generic repositories where they are more visible.

## Conclusion: managing data-sharing to facilitate data reuse

The world of research and scientific publishing is moving and evolving at a rapid pace. A decade ago peer-reviewed publications were viewed as the single mechanism for the dissemination of experimental results. Today, researchers can inform and share their research via a multitude of platforms such as blogs, open notebooks, wikis, and even Twitter. Data are no longer a by-product of research to be summarized in a table within a paper or stored in a laboratory notebook on a shelf. They are becoming a measurable output of research in its own right. Researchers, funders, and governments are recognizing the value of data not only to the scientist that generated it but also in its reuse by others who may wish to exploit these data to further additional investigations and discoveries (Leonelli, 2013).

In this paper, the storing and sharing of three of the major ‘-omics’ data types (transcriptomics, proteomics, metabolomics) was assessed as well as the possible avenues for their storage and dissemination. Despite the popularity enjoyed by these data types, which are amongst the best known and most commonly generated in contemporary biology, there is not a single, overarching model for how they should be curated and disseminated in databases. It is not clear whether such a unified model is possible or even recommendable, given the diversity of challenges attached to the interpretation of these data. What is clear, however, in light of our review of available mechanisms for data curation and release, is that scientists are in the best position to assess and tackle this problem. Researchers at the bench have the highest degree of familiarity with data production and they are the ones ultimately responsible for data reuse. This is clearly illustrated by some of the most utilized databases, which were originally generated by specific groups of scientists to answer a specific problem within their own research and have ended up being beneficial to a much wider audience.

We do not wish to suggest that each research group/project should generate its own databases with its own terminology and data formats. Rather, it is argued that, where possible, data should be curated and formatted according to community-agreed standards and stored in internationally recognized repositories. For data types such as metabolomics, where community standards are not well established and there is no defined repository, two possible platforms for dissemination have been discussed: those provided by publishers and those provided by universities. Repositories provided by publishing houses provide a useful and clear mechanism to ensure that the underlying data is always linked to a paper; and in the case of data journals, they even provide a way to publish datasets in their own right. However, they do not provide an adequate solution to the problem of database interoperability and standards, and the same is also true of university repositories. University repositories have been viewed by some funders and governments not only as a useful mechanism to solve the data-storage problem but also as means to solve another pressing issue with databases, i.e. their longevity and sustainability in the long term. However, there is no guarantee that repositories hosted within a university will not be restructured or destroyed to follow latest policy/market requirements; and IT solutions devised without an eye for individual and disciplinary variation will struggle to cope with the specific needs and requirements of data producers and users. In light of these caveats we would suggest that repositories provided by publishing houses and universities are only utilized as interim or additional data stores, whilst community-generated solutions and standards are created. Without such community-driven solutions it is not possible to provide well-curated data that is of a high enough standard to make it possible to automate searches (machine readability) and also provide efficient ways to trace provenance so that it is intelligible to researchers (human readability).

The issues surrounding data storage, access, and reuse are extremely complex and the solutions that are needed will vary considerably depending on field, type of data, and scientific goals. At present, a large number of scientists are not motivated to share data because of a lack of recognition and reward and also not having sufficient time, expertise, and resources to devote to the task of data curation. Transforming the situation will require incentives from publishers, funders, industry, and universities for data-sharing and reuse that is community driven; as the most successful data-sharing initiatives come from scientists themselves. There also needs to be a transformative change in how the efforts put into data curation and dissemination are rewarded by institutions and how they are valued by the community.

It is concluded that community and project-specific databases are the most useful to researchers interested in effective data-sharing since these databases are explicitly created to meet the researchers’ needs, support extensive curation, and embody a heightened awareness of what it takes to make data reusable by others. Such bottom-up and community-driven approaches need to be valued and provided with long-term sustainable support by funding bodies and publishers alike. At the same time, these databases need to be linked to generic databases where possible, in order to be discoverable to the majority of researchers and thus promote effective and efficient data-sharing. As we look forward to a future that embraces open access to data and publications, it is essential that data policies, data curation, data integration, data infrastructure, and data funding are linked together into a single continuum that is centred on data access and research productivity.
